# Anatomic Reconstruction of the Distal Radioulnar Ligament for Posttraumatic Distal Radioulnar Joint Instability

**DOI:** 10.4055/cios.2009.1.3.138

**Published:** 2009-08-17

**Authors:** Kyu Nam Seo, Min Jong Park, Hong Je Kang

**Affiliations:** Department of Orthopedic Surgery, Samsung Medical Center, Sungkyunkwan University School of Medicine, Seoul, Korea.

**Keywords:** Distal radioulnar joint, Instability, Ligament reconstruction

## Abstract

**Background:**

To analyze clinical outcomes after anatomical reconstruction of distal radioulnar ligaments in patients with chronic post-traumatic instability of the distal radioulnar joint.

**Methods:**

Anatomical reconstruction was performed in 16 patients with subluxation or dynamic instability of distal radioulnar joint following trauma. Osteotomy was performed simultaneously in 10 patients with radial malunion. The average follow-up period was 18.9 months. For clinical outcome assessment, we performed the anteroposterior stress test, measured the range of motion and grip strength, and performed radiological examination. For assessment of the pain and function, we used the Patient Rated Wrist Evaluation, the Disabilities of the Arm, Shoulder and Hand, and the Modified Mayo Wrist Score.

**Results:**

Anteroposterior stress test performed at the last follow-up showed normal in 12 patients, mild laxity in 3, and residual subluxation in one. The average Patient Rated Wrist Evaluation was 9.1 for pain and 11.2 for function. The average Disabilities of the Arm, Shoulder and Hand score was 10.5. The average Modified Mayo Wrist Score was 92.8; there were 10 excellent, 5 good, and 1 poor case. The average grip strength improved from 69.7 1b to 80.9 1b. A revision osteotomy was performed on the patient with residual subluxation in order to obtain normal alignment of the joint.

**Conclusions:**

Anatomical reconstruction of the distal radioulnar ligaments is recommended to restore distal radioulnar joint stability. In addition to ligament reconstruction, realignment of the distal radioulnar joint seems critical when the instability is combined with malunion of the radius.

Stability of the distal radioulnar joint (DRUJ) is provided by bony architecture and by soft tissues such as the triangular fibrocartilage complex (TFCC), the joint capsule, and surrounding muscles.1) Cadaver studies have demonstrated that the volar and dorsal distal radioulnar ligaments, which are components of the TFCC, play a major role in stabilizing the joint.^2^-5)

Many authors have made various attempts to restore DRUJ stability surgically following trauma. Some have employed indirect methods such as tenodesis using the flexor carpi ulnaris tendon or the extensor carpi ulnaris tendon.^6^-8) However, these nonanatomic procedures are not reliable in that the normal stability of the joint is not restored and the joint motion may be restricted. Anatomic reconstruction of the major structures responsible for joint stability is the most important principle for the treatment of instability of an injured joint with an intact articular surface. In this sense, some authors have tried to achieve anatomic reconstruction of the volar and dorsal distal radioulnar ligaments for DRUJ stability. Scheker et al.9) reconstructed the ligaments in 15 patients by using a tendon graft whose ends were anchored in pre-drilled holes in the radius and the ulna. Adams and Berger10) reported the clinical results of a procedure in which a tendon graft was passed through near the normal anatomic attachment site of the volar and dorsal distal radioulnar ligaments to attempt the best possible anatomic reconstruction.

The objective of this study was to analyze clinical outcomes after anatomic reconstruction of the distal radioulnar ligaments in patients with posttraumatic chronic instability of the DRUJ. The study was based on the idea that the best outcomes can be obtained with anatomic reconstruction of the soft tissue stabilizers and realignment of the DRUJ.

## METHODS

### Patients

Sixteen patients with posttraumatic DRUJ instability underwent anatomic reconstruction of the distal radioulnar ligaments between February 2002 and June 2006. Subluxation during forearm rotation or dynamic instability based on the anteroposterior stress test was observed in all patients.

The anteroposterior stress test was performed with the forearm in neutral position. The examiner firmly grasped the radius with one hand, held the ulnar head with the other hand, and applied anteroposterior force. Depending on the symptoms experienced by the patient and the side-to-side difference in translation, the test results were classified into one of four grades. Grade 0 was defined as normal stability, Grade I as ligamentous laxity without functional impairment, Grade II as dynamic instability, and Grade III as spontaneous subluxation and reduction during active forearm rotation (Table 1). Surgical reconstruction was indicated in Grade II and III patients. The patients who showed damage to the articular surface of the DRUJ observed on plain radiographs or computerized tomography, irreducible subluxation of the joint, and significant joint stiffness were excluded.

**Fig. 1:**
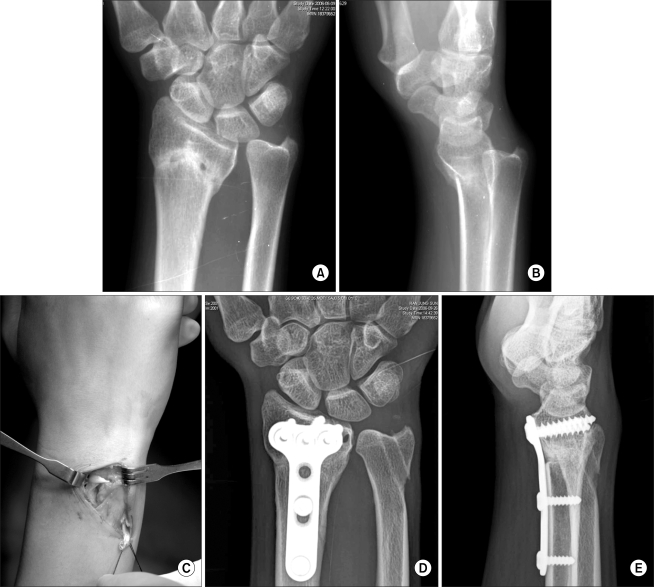
Preoperative radiographs of a 24 year-old woman shows distal radioulnar joint subluxation associated with radial malunion (A & B). This patient was treated with osteotomy and distal radioulnar ligament reconstruction using the palmaris longus tendon (C). Radiographs obtained two years after surgery show restored alignment. Note that the bone tunnels for ligament reconstruction in the distal radius and ulnar head are visible (D & E).

There were 11 male and 5 female patients with an average age of 26.9 years (range, 15 to 57 years). The mean follow-up period was 18.9 months (range, 12 to 38 months). Eleven patients had volar subluxation or instability and 5 had dorsal subluxation or instability. Six patients underwent distal radioulnar ligament reconstruction as a single procedure. The presumed causes of DRUJ instability were TFCC peripheral tear in four patients and ulnar styloid base fracture in two patients (Table 2). In 10 patients, instability was also caused by malunion of a radial fracture (distal radial metaphyseal fracture in 5, distal radial shaft fracture in 2, mid-shaft radius fracture in 2, radius and ulna shaft fractures in 1). Corrective osteotomy was performed in addition to distal radioulnar ligament reconstruction (Table 3) (Fig. 1). The osteotomy was created in the distal radius in 5 patients and in the radial shaft in 4 patients. Ulnar shortening was performed in 1 patient who had radial shortening following a distal radial metaphyseal fracture. For internal fixation, plates and screws were used in 9 patients, and K-wires were used in a 15-year-old patient.

### Surgical Technique^10^,11)

A 4 to 5 cm transverse skin incision was made from the distal ulnar head extending proximally along the fifth extensor compartment. The 5th extensor compartment was opened, and the extensor digiti minimi tendon was retracted radially. An L-shaped flap was created in the DRUJ capsule, with one limb made along the dorsal rim of the sigmoid notch and the other made proximal and parallel to the dorsal DRUL.

A palmaris longus tendon graft was harvested, and each end of the graft was connected to a thread to facilitate easy passage through the tunnels. The periosteum was dissected from the distal and dorsal margin of the sigmoid notch to make an anteroposterior tunnel in the radius. A guide wire was inserted in parallel with the articular surface of the sigmoid notch. The proper guide wire position was checked with a C-arm followed by creation of a 3-4 mm tunnel for the graft without damaging the subchondral bone of the radiocarpal joint and the sigmoid notch. A 2 cm transverse incision was made over the volarly protruding guide wire to expose the volar opening of the tunnel and the volar side of the DRUJ. A 2.0 mm drill hole was made in the radius, then gradually enlarged with a straight curette to allow for passage of the tendon graft. To make the ulnar tunnel, the wrist was flexed volarly and the TFCC was pulled distally to expose the ulnar fovea. With the ulna pulled dorsally, a tunnel was made through the ulnar fovea at a tilt angle toward the ulnar side of the ulnar neck in the same way for the radial tunnel. The tendon graft was passed through the radial tunnel and both of its limbs were brought to the opening in the ulnar fovea. In bringing the volar limb of the tendon graft to the ulnar fovea, a hemostat was used to pierce the anterior capsule between the ulnar head and the TFCC without damaging the ulnar nerves or vessels. Then, both limbs were passed through the ulna tunnel to the ulnar side of the ulnar neck. The limb on the volar side surrounded the ulna and was sutured with the other limb using 3-0 nonabsorbable sutures (Fig. 1C). During the suturing procedure, both limbs were pulled taut with the forearm in neutral position, and care was taken not to restrict forearm rotation by imposing excessive tension. Motion of the wrist joint and the DRUJ was checked, and the final status of the reconstructed tendon was assessed with the anteroposterior stress test.

In patients with radial malunion, a planned osteotomy was performed prior to distal radioulnar ligament reconstruction to restore normal DRUJ alignment. After proper internal fixation was applied, an anteroposterior stress test was performed to determine the necessity of distal radioulnar ligament reconstruction. Because instability was still observed in all patients, distal radioulnar ligament reconstruction was performed at the same time. In one patient with radial shaft malunion, distal radioulnar ligament reconstruction actually preceded osteotomy because the preoperative angular deformity on the sagittal plane was not definite, but subluxation during supination was observed requiring additional distal radius osteotomy.

Postoperatively, long-arm cast was applied for 6 weeks, followed by range of motion exercise to restore normal joint mobility. Normal activity was allowed at 10-12 weeks postoperatively, once normal range of motion was achieved.

### Evaluation

Objective clinical examination was performed using the anteroposterior stress test, range of motion evaluation, grip strength assessment using a Jamar dynamometer (Jamar; Smith and Nephew, Memphis, TN, USA), and plain radiography. Subjective clinical evaluation was performed using the Patient Rated Wrist Evaluation (PRWE),12) the Disabilities of the Arm, Shoulder and Hand (DASH),13) and the Modified Mayo Wrist Score.14)

The PRWE is a 15 item questionnaire used to assess wrist pain (5 items) and function (10 items: 6 for specific activities and 4 for usual activities). Each score ranges from 0 (no pain or difficulty in activities) to 10 (severe pain or being dysfunctional). The DASH is a 30 item self-report questionnaire designed to evaluate the function and symptoms of the upper limb regardless of surgical site. Answers to questions about daily activities are ranged from 0 (no impairment) to 100 (maximum impairment). The Modified Mayo Wrist Score is composed of four sections (pain, level of activity, range of motion, and grip strength), with the total score rated as excellent (90-100), good (80-90), fair (65-79), or poor (under 65).

For 10 patients who underwent osteotomy in addition to distal radioulnar ligament reconstruction, preoperative and postoperative changes in the radial angle were observed in the sagittal plane. The absolute values of the side-to-side difference in angle were recorded. The volar tilt angle was measured in patients with an osteotomy of the distal radius, and the angle at the longitudinal axis of the shaft was measured in those with an osteotomy of the shaft. The ulnar variance and radial inclination were also measured and compared.

### Statistical Analysis

Preoperative and postoperative values were analyzed using the Wilcoxon signed rank test. The SPSS ver. 10.0 program was used for statistical analysis, with the level of statistical significance set at *p* < 0.05.

## RESULTS

### Assessment of Instability

**Fig. 2:**
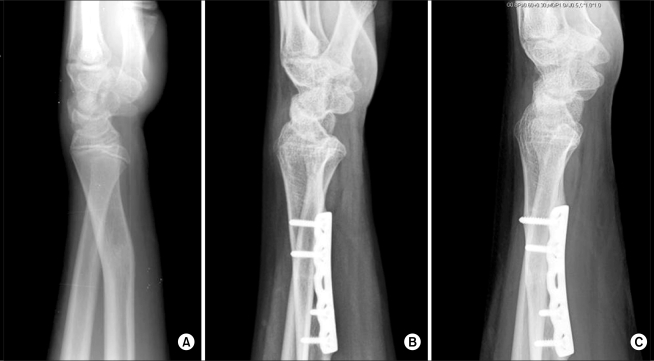
A 15 year-old boy with volar subluxation of the ulnar head following radial malunion (A) had been treated with osteotomy and distal radioulnar ligament reconstruction. Residual instability was present due to insufficient restoration of distal radioulnar joint alignment (B). Redo-osteotomy improved the alignment, resulting in subsidence of subluxation with mild laxity (C).

The anteroposterior stress test performed at the last follow-up visit showed no side-to-side difference in 12 patients and laxity with a firm end point (Grade I) in 3 patients. In one patient, reoperation was required because subluxation was noted during rotation (Grade III). The patient was a 15-year-old male who had a history of cast immobilization due to fracture of the shaft of the radius incurred while skiing 14 months prior to operation. Physical examination demonstrated that the ulnar head was volarly subluxated in supination and reduced in pronation. Radiographs showed 15° of volar angular deformity in the radial shaft. After closed wedge osteotomy of the radial shaft and distal radioulnar ligament reconstruction, subluxation improved but still remained. On postoperative radiographs, correction of the angular change was insufficient, especially on the sagittal plane. We attributed the persistent subluxation to the failure to restore the normal alignment of the DRUJ. We performed a second corrective osteotomy 18 months later. Unlike the first osteotomy, the second procedure involved open osteotomy and bone graft to achieve sufficient angular correction. On the anteroposterior stress test performed at the last follow-up visit, the patient's instability was classified as Grade I (Fig. 2).

### Range of Motion

The average range of extension in the wrist joint was 76.9° (range, 30 to 90°) preoperatively and 72.8° (range, 60 to 80°) postoperatively. The average range of flexion was 69.7° (range, 40 to 90°) preoperatively and 70.9° (range, 50 to 90°) postoperatively. The average ulnar deviation was 29.1° (range, 20 to 40°) preoperatively and 30.3° (range, 25 to 60°) postoperatively. The average radial deviation was 15.0° (range, 10 to 40°) preoperatively and 13.4° (range, 10 to 20°) postoperatively. There was no statistically significant difference between the preoperative and postoperative ranges of rotation. Regarding the forearm rotation, the average range of pronation changed from 76.6° (range, 45 to 90°) to 76.3° (range, 70 to 90°), while the average range of supination changed from 83.8° (range, 70 to 90°) and 82.5° (range, 70 to 90°), indicating no significant difference. No patients showed greater than 10° of decrease in the range of rotation (Table 3).

### Grip Strength

The average grip strength significantly increased from 69.7 1b (range, 40 to 120 1b) preoperatively to 80.9 1b (range, 40 to 130 1b) postoperatively (Table 3) (*p* = 0.006).

### Radiological Examination

Based on the radiological findings in 10 osteotomy patients, the side-to-side differences in the sagittal plane were significantly reduced from 19.0° (range, 6 to 39°) preoperatively to 4.8° (range, 2 to 10°) postoperatively (*p* = 0.005) (Table 3).

### PRWE, DASH and Modified Mayo Wrist Score

The mean PRWE pain score (0 = maximum, 50 = minimum) improved significantly from 23.1 points (range, 0 to 45 points) preoperatively to 9.1 points (range, 0 to 34 points) postoperatively, while the mean PRWE functional score (0 = maximum, 100 = minimum) improved from 39.2 points (range, 5 to 69 points) preoperatively to 11.2 points (range, 0 to 48 points) postoperatively. The mean DASH score (0 = maximum, 100 = minimum) improved from 34.5 points (range, 5.8 to 56.7 points) preoperatively to 10.5 points (range, 0.8 to 40.8 points) postoperatively. The Modified Mayo Wrist Score (100 = maximum, 0 = minimum) also improved from a preoperative mean of 72.5 points (range, 25 to 95 points) to a postoperative mean of 92.8 points (range, 70 to 100 points); 10 patients were rated as excellent, 5 were rated as good, and one patient who had undergone reoperation due to residual subluxation were rated as poor just before the second osteotomy was performed. Statistically significant advancement was noted on the preoperative and postoperative comparison of subjective pain and function (*p* < 0.05) (Table 3).

## DISCUSSION

The DRUJ is formed by the sigmoid notch of the radius and the ulnar head. It is responsible for the forearm rotation, one of the most important functions of the upper arm. The morphological character of the joint allows for simultaneous rotation and anteroposterior translation. The role of the bony architecture in joint stability is limited, and inherent anteroposterior instability is inevitable. The soft tissue structures contribute significantly to the stability of the joint. Effective treatment of joint instability should be based on studies of normal anatomy and mechanics. Since Palmer and Werner4) described the structure of the TFCC in 1981, many authors have shown in their cadaver studies that the volar and dorsal distal radioulnar ligaments of the TFCC are the key structures in joint stability.^1^,^2^,5) Accordingly, the methods for treating DRUJ instability have evolved from nonanatomical methods such as tenodesis to anatomical methods such as reconstruction of the distal radioulnar ligaments. As anatomical reconstruction has been proven as the optimal treatment for instability in other joints such as knee and elbow, this attempt is quite natural. Considering that the reconstruction procedures suggested by Adams et al.^10^,15) using the palmaris longus tendon has been shown to be the most reliable and anatomically acceptable surgical technique, we performed distal radio ulnar ligament reconstruction according to their method.

DRUJ instability is difficult to diagnose because it presents with variable and often indistinct symptoms and lacks diagnostic criteria. The diagnostic criteria of DRUJ instability in the current study included repeated subluxation and reduction or dynamic instability observed during the stress test. Subluxation developed in patients with DRUJ malalignment as a result of radial malunion. Subluxation occurs when the ulnar head is relatively anterior or posterior to the sigmoid notch of the radius and is directly associated with the angular deformity of the radius in the sagittal plane. For these patients, corrective osteotomy is essential because malalignment of the DRUJ is the basic mechanism of instability.16) In the 10 patients who underwent osteotomy in this study, angular formations ranging from 6° to 40° were observed from the distal radius to the proximal shaft. Theoretically, the likelihood of joint instability increases, the more proximal the location of an angular formation is when the patients have same angular formation, and the greater the angular formation is when the patients have the same location of an angular formation. However, DRUJ instability was also observed in patients with generally acceptable degrees of malunion of the distal radius, indicating that the development of joint instability is also attributed to damage to the distal radioulnar ligaments.

DRUJ reconstruction is not mandatory in patients with malunion of the radius because instability can improve when normal alignment of the joint is achieved by corrective osteotomy.^16^,17) In this study, although the osteotomy resulted in some improvement, the anteroposterior stress test after osteotomy still revealed Grade II instability. This indicates that satisfactory results cannot be expected only by the joint realignment in patients with obvious subluxation because the dynamic instability would impair the DRUJ function. It is clear that ligament reconstruction is required when functional disability is largely attributable to dynamic instability rather than malunion. We believe that when the major pathology is diagnosed as DRUJ instability in a patient with malunion of the radius, it is desirable to perform osteotomy and ligament reconstruction simultaneously.

Excluding the one patient who underwent a second reconstruction surgery, the postoperative anteroposterior stress test showed the joint instability within the normal range. Functional results were satisfactory with preservation of joint motion. Joint laxity which means increased anteroposterior translation should be regarded as normal if an obvious end point is observed and no pain or apprehension is present in the anteroposterior stress test. With the exception of one patient, satisfactory postoperative improvements were also obtained in subjective evaluation.

In the patient with the unsuccessful results, volar subluxation of the ulnar head during supination improved but still remained following corrective radial osteotomy and ligament reconstruction. The angular malunion of the radius seemed not to be corrected enough on the plain lateral radiograph; too much attention might have been directed to radial shortening during the closed osteotomy. Following the second corrective osteotomy, we observed grade I instability in the intraoperative anteroposterior stress test. We thought that the prior anatomical ligament reconstruction provided enough stability, requiring no revision of ligament reconstruction. At the last follow-up, no objective symptoms or signs of subluxation were noted, but the patient was not satisfied with the outcome and complained of mild pain, weakness, and functional impairment.

Anatomical reconstruction of the distal radioulnar ligaments is thought to be an effective procedure for treating posttraumatic DRUJ instability. In addition to ligament reconstruction, osteotomy should be performed for anatomical alignment of the DRUJ when the instability is accompanied by malunion of the radius.

## Figures and Tables

**Table 1:**
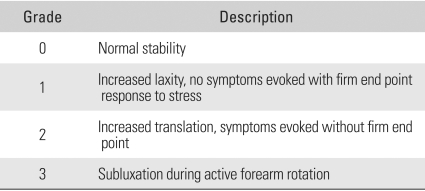
Grades of Distal Radioulnar Joint Instability during Anteroposterior Stress Test

**Table 2:**
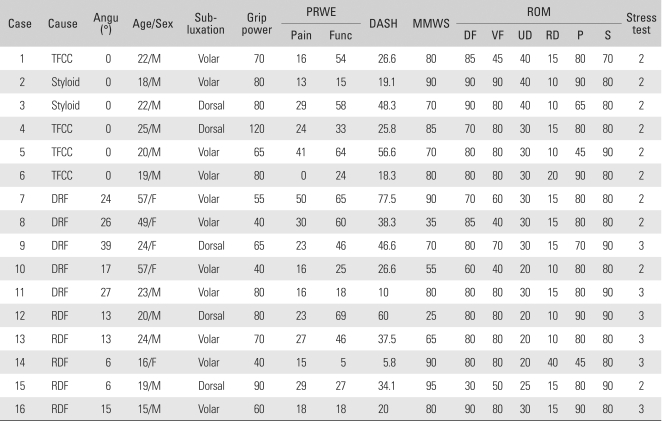
Preoperative Demographics of All Patients

Angu: Angulation, PRWE: Patient rated wrist evaluation, DASH: Disabilities of the arm, shoulder, and hand, MMWS: Modified Mayo wrist score, ROM: Range of motion, DF: Dorsiflexion, VF: Volar flexion, UD: Ulnar deviation, RD: Radial deviation, P: Pronation, S: Supination, TFCC: TFCC peripheral tear, Styloid: Ulnar styloid base fracture, DRF: Distal radius fracture, RDF: Radius diaphysis fracture.

**Table 3:**
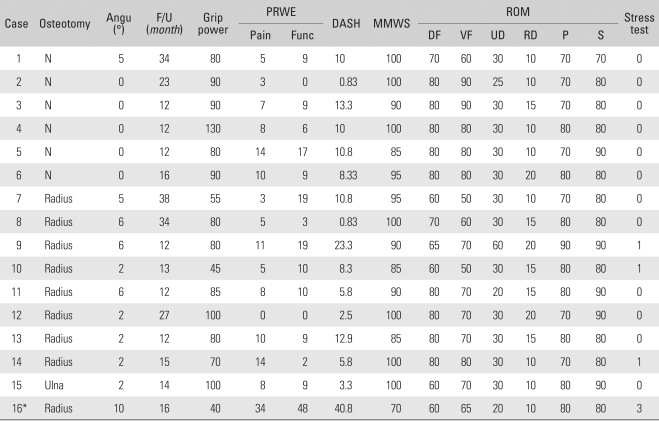
Surgical Treatments and Postoperative Outcomes

^*^Instability symptoms during follow-up. Second corrective osteotomy was done. Angu: Angulation, PRWE: Patient rated wrist evaluation, DASH: Disabilities of the arm, shoulder, and hand, MMWS: Modified Mayo wrist score, ROM: Range of motion, DF: Dorsiflexion, VF: Volar flexion, UD: Ulnar deviation, RD: Radial deviation, P: Pronation, S: Supination.
